# Telemonitoring with respect to Mood Disorders and Information and Communication Technologies: Overview and Presentation of the PSYCHE Project

**DOI:** 10.1155/2014/104658

**Published:** 2014-06-24

**Authors:** Hervé Javelot, Anne Spadazzi, Luisa Weiner, Sonia Garcia, Claudio Gentili, Markus Kosel, Gilles Bertschy

**Affiliations:** ^1^INSERM U1114, Fédération de Médecine Translationnelle de Strasbourg, Université de Strasbourg, Pôle de Psychiatrie et Santé Mentale des Hôpitaux Universitaires de Strasbourg, 67000 Strasbourg, France; ^2^Service des Spécialités Psychiatriques, Département de Santé Mentale et de Psychiatrie, Hôpitaux Universitaires et Université de Genève, 1205 Genève, Switzerland; ^3^Dipartimento di Patologica Chirurgica, Medica, Molecolare e dell'Area Critica, Università di Pisa, 56126 Pisa, Italy

## Abstract

This paper reviews what we know about prediction in relation to mood disorders from the perspective of clinical, biological, and physiological markers. It then also presents how information and communication technologies have developed in the field of mood disorders, from the first steps, for example, the transition from paper and pencil to more sophisticated methods, to the development of ecological momentary assessment methods and, more recently, wearable systems. These recent developments have paved the way for the use of integrative approaches capable of assessing multiple variables. The PSYCHE project stands for Personalised monitoring SYstems for Care in mental HEalth.

## 1. Introduction

Mood disorders are associated with high levels of morbidity and mortality, and they also engender enormous costs in society because of their high prevalence, early onset, and episodic course [1, 2]. According to the “global burden of disease study,” mood disorders are a major concern for public health and will continue to be in the years to come; the burden they represent is comparable to that associated with ischemic heart diseases or respiratory tract infections [3].

The estimated burden associated with mood disorders is essentially due to major difficulties with patients' care, namely, that pharmacological treatments usually have week-long onset latencies, and it is impossible to anticipate individual responses to treatment. Moreover, a substantial proportion of patients have relapses during their lifespan. It is also well known that compliance with drug treatments and specific lifestyle changes (social functioning and biological rhythms) are important issues for the long-term management of mood disorders.

Some studies have identified the importance of sleep, stress, and other modifiers of daily routine in mood disorders [4–8]. However, rather than revealing the causes of mood variations, sleep disturbances, or circadian rhythm modifications, these studies merely show how they are interrelated and interact with each other. Event-recording apparatus, such as mood or asleep/awake logs, is for the most part subjective since such logs are recorded by the patients themselves and are generally unrelated to more objective data (e.g., physiological parameters). New technical developments in the field of wireless intelligent sensors, such as wearable health systems which allow for ambulatory multiparametric monitoring, have benefited from extensive evaluation over the past decade [9–20].

In the same context, the European PSYCHE project (Personalised monitoring SYstems for Care in mental HEalth) has developed an interactive system based on the long-term recording of physiological and clinical parameters with user-friendly portable recording devices (smart fibers and interactive textile) and a smartphone for the outpatient environment [21–23].

We present the PSYCHE project below and start by reviewing evidence with respect to objective markers in mood disorders (e.g., clinical, biological, and physiological markers). Then we describe how the use of information and communication technologies has developed in relation to mood disorders, right up to the development of wearable systems, and, more recently, integrative approaches allowing for the assessment of multiple variables.

## 2. Objective External Markers of Patients' Clinical State and Outcome Prediction in Mood Disorders

### 2.1. Clinical Issues and Biological Factors

An “optimal” care approach to mental health involves the prediction of treatment response and the risk of relapse. Many great parameters, such as the clinical course, have been identified as potential predictors. For instance, the number of previous episodes and subclinical residual symptoms affect the risk of relapse in depression and bipolar disorder [24–26]. Stressful life events, shorter intervals between episodes, and the persistence of affective symptoms may also precipitate relapse in bipolar illness [24, 26]. For instance, according to Kleindienst et al. [27] a favourable response to lithium could be associated with an episodic pattern of mania-depression-interval and onset of the illness at an older age, whereas a negative response to lithium seems to be related to a high number of previous hospitalizations, an episodic pattern of depression-mania-interval and rapid-cycling mood episodes.

In addition to clinical factors, biological and neuroimaging data are also important sources of information for prediction purposes [28–34]. However, although they provide crucial information for understanding psychiatric disorders, their use is limited because of their cost and their limited sensitivity and/or specificity. For the time being, generalization of these approaches beyond the realm of fundamental research remains uncertain. In the genetics field, for instance, the use of polymorphisms is insufficient to justify personalized prescription, and more elaborate models are needed [35–37].

### 2.2. Objective Parameters in Bipolar Disorders and Their Use in Clinical Psychiatry

Objective measurements are rare with psychiatric conditions and are of very limited use in clinical settings. For instance, the most accurate correlates of mental disorders can be drawn from neurobiological studies involving electroencephalographic (EEG), functional magnetic resonance imaging (fMRI), or positron emission tomography (PET). These techniques are expensive and difficult to use on a regular clinical basis. Other measurements and variables, however, were also taken in consideration. For instance, poorer physical functioning and increased motor activity are associated with subclinical depressive and manic symptoms, respectively, and could predict relapses in these two clinical states [38–43]. However, Burton et al.'s review [44] of monitoring of depression highlights the lack of information stemming from controlled and longitudinal studies that can be used to (i) determine the level of activity in healthy subjects, (ii) establish correlations with other validated measures, and (iii) determine the acceptability of monitoring systems. Moreover, there would appear to be a need for efficient and standardized analytical methods for extracting relevant information from actigraphy data and for distinguishing different subtypes of mood disorder recordings [44, 45].

Heart and respiratory rates are also potential markers for stress, sleep quality, and sleep/wakefulness transitions. The monitoring of heart rate variability (HRV) can serve to evaluate the mutual influence at play between the central and peripheral nervous systems and could be a promising way of tracking down mood variations in psychiatry [46–48]. Although software exists for analyzing cardiac coherence, it is a technique which requires the continuous measurement of heart rate by electrocardiography (ECG), making its long-term use difficult.

EEG activity could be also an appropriate tool, especially for predicting the therapeutic response in depression [49] and bipolar disorder [50], but again such a strategy is considered difficult to implement in the context of routine monitoring. Moreover, EEG is affected by numerous artefacts [51] and their corrections (e.g., eye tracking for ocular movements [52]) can lead to even weightier methods that are difficult to implement in routine clinical practice.

Plethysmography is a noninvasive technique used to assess vascular functioning and to obtain complex cardiorespiratory data [53]. It is a detection method used notably in the LifeShirt system [53, 54] and seems to be a useful monitoring tool, especially for psychiatric disorders involving severe physiological disturbances such as panic disorder [53, 55, 56]. Its variant, photoplethysmography, is generally used with an optical device attached to one finger of the nondominant hand, but other devices such as glasses could be less obtrusive and generate less artefacts than those attached to the finger [57, 58]. On the whole, photoplethysmography still serves solely as an alternative way of measuring HRV, with no definite advantages [57].

Electrodermal activity (EDA) records biological electrical activity on the surface of the skin and is a technique known since the late 19th century. EDA reflects the activity of the autonomic nervous system, and EDA hyporeactivity has been described in many studies of depressive patients, but very little data is available with respect to bipolar patients [59]. In a recent study, bipolar patients showed the highest prevalence of electrodermal hyporeactivity compared to patients with other affective disorders [59]. Preliminary results [60] showed that different mood states can be related to EDA.

There is strong evidence in the literature of a link between dysregulation of the circadian rhythms and affective disorders [4, 5, 7, 8]. More precisely, motor activity rhythm, sleep/wake cycles, sleep architecture, thermoregulation, and hormonal cycles (e.g., cortisol and melatonin) seem to be disturbed in bipolar disorder [8]. The impairment observed in bipolar patients is mainly a phase advance of these circadian rhythms [8, 40]. Moreover, in subjects with bipolar disorder their quality of life perception and their ability to pursue valued activities and interests are compromised. In addition, according to the zeitgeber social theory, depressive episodes may be triggered by life events that disrupt social rhythms, which, in turn, derail physiological homeostasis or the regularity of circadian rhythms [61, 62]. Sleep disturbance is a key symptom of mood disorders [6]. Whereas insomnia and hypersomnia may occur during major depression episodes, a reduced need to sleep is closely related to manic episodes [63]. Body temperature is known to be negatively correlated with plasma melatonin levels, and desynchronization of the neurohormone cycle in psychopathological contexts can disrupt temperature regulation [64]. These disruptions are well characterized during depression and depressive states of bipolar disorder [64, 65]. However, rectal temperature, which remains the reference measurement, is too invasive, and, more generally, temperatures in the normal range are hard to interpret because of the variations linked to gender, age, and biological rhythms [66, 67].

The relationship between mood and certain physical variables extracted from speech is also well established. Depressed speech, for instance, is traditionally defined as dull, monotone, monoloud, and lifeless [68]. These perceptual characteristics have been associated with acoustical parameters involving fundamental frequency, amplitude modulation, formant structure, spectral power distribution, pause frequency and duration, and jitter [69–72]. Recent data obtained from a study that focused on voice acoustic parameters as biomarkers of depression severity and treatment response showed that depressed patients present longer speech pause times, which tend to shorten with clinical improvement in treatment responders [73]. In bipolar disorder, whereas the variation in speech fluency is well established [74, 75], there is a paucity of data relating to the acoustic properties associated with pressured speech during manic episodes. To assess the acoustic characteristics associated with the different phases of the bipolar conditions better, the critical step remains the construction of algorithms establishing correlations between speech analysis and clinical observations [76].

Among these physiological parameters, heart and respiratory rates, nocturnal sleep, daytime activity measured by actigraphy, and voice analysis seem to be the most promising in terms of monitoring in clinical settings.

The aforementioned studies, which focused on specific clinical or physiological aspects, show the limits of our knowledge of the biological counterpart of mood disorders, and such limited knowledge also has consequences for our ability to estimate and predict the clinical course of bipolar disorders. This is an observation, however, which specifically applies to studies that consider one physiological variable at a time and which thus argues in favour of using a multivariable approach. To achieve such a goal, there is a need for a multiparametric monitoring system that is easy to use in the clinical follow-up of patients with mood disorders. Technological progress towards the miniaturization of electronic devices, which become portable, and their integration in everyday objects such as items of clothing or mobile phones can contribute to achieving this aim. New-generation systems allow for devices that are more user friendly and more manageable and which can be used for long-term and long-lasting monitoring. Although this new kind of “remote medicine” has existed for more than 20 years, over the past five years it has experienced rapid development with the use of wellness wearable system (WWS) in many medical fields [16, 18, 20, 77–79].

## 3. Information and Communication Technologies (ICTs) in Mood Disorders

### 3.1. From Paper and Pencil Methods to ICT-Based Solutions

The use of ICTs in the field of health has increased considerably in recent years. Computers and the Internet offer new possibilities for monitoring patients. These developments provide an incentive to rethink the organization of healthcare, with patients moving from a passive to an active role [80]. In psychiatry, this transition has received support from, inter alia, the European institutions which have called for the development of ICT-based solutions for people with mental illness, through multidisciplinary partnerships [81].

Optimum management of patients with mood disorders would include continuous assessment of their mood swings. Several paper-based mood charting devices (e.g., Life Chart Methodology [LCM], STEP-BD [Systematic Treatment Enhancement Program for Bipolar Disorder] Mood Chart, and Chronosheet) are currently used as self-reporting methods for following the clinical course of patients with bipolar disorder and for promoting their self-management skills [81]. The transition from the paper-based Chronosheet mood charting to the ChronoRecord software is a good example of how the first wave development of ICTs can be put to good use in clinical practice [81]. Paper-based mood charting is associated with low compliance, and lengthy transcriptions of the data are needed for research purposes. Today, the development of comprehensive tracking of daily mood directly on the patient's personal computer seems feasible, because so many households are equipped with a computer due to the widespread acceptance of computer technology. ChronoRecord is an example of user-friendly software that is used to measure a significant number of data collected daily (mood, sleep, medication, life events, and menstrual cycle for female) or weekly with a high level of patient adherence [82, 83]. Two studies have demonstrated concurrent validity of self-reporting with the ChronoRecord software and clinician ratings using the Hamilton Depression Rating Scale (HAMD) and the Young Mania Rating Scale (YMRS) [83, 84]. Furthermore, although a potentially serious bias could be related to the ability of people to use a computer, another study showed that age, years of education, gender, diagnosis, and disability status did not seem to bias patients' use of the ChronoRecord software [85]. A similar study that compared a paper version of the LCM and an online version also demonstrated that patients filled in the electronic chart more often and more carefully than the paper chart [86].

### 3.2. The Development of Wearable Systems

#### 3.2.1. Ecological Momentary Assessments

There are two types of procedures with real-world and real-time assessment, usually referred to as “ecological momentary assessment” (EMA) and “experience sampling methodology” (ESM). The first focuses on phenomenological experience, whereas the second integrates objective data such as physiological parameters [87].

Although “paper-pen” assessment may be accompanied by alarm devices, adherence to such procedures remains low [88]. That is why the use of new technologies such as smart phones, personal digital assistants, handheld computers, or digital tablets appears to be useful for increasing patient adherence for data collection [87]. For the moment, the integration of electronic technologies in momentary assessment has benefited the assessment of depressive disorders more than that of bipolar disorders [89, 90].

#### 3.2.2. Wearable Health Systems

Wearable health systems are already available as means of enhancing health care and combine biomedical technologies, microtechniques, and communication technologies. As Lymberis and Gatzoulis showed in their review [91], it is possible to develop personal health monitoring systems. With their heightened reliability and ability to record different parameters objectively to a greater degree of precision, these new technologies will pave the way for new investigative methods which will produce answers beyond the scope of those used in older studies.

As of today, studies increasingly make use of high technology to record data and enhance care. The study by van den Berg et al. [92] developed a treatment for patients with mental disorders in rural regions that relies on a telemedical care concept based on telephone contacts and text messages, while the review by Ehrenreich et al. [93] emphasizes how mobile technologies can enhance the delivery of psychiatric interventions. Other studies have targeted the management of bipolar disorder from a psychoeducational perspective through the use of mobile phone technology [94, 95]. Recent studies also show that encouraging experiments are being conducted in the field, enabling remote care a long way from hospitals and the development of so-called “mobile psychiatry” [96–98] which refers to personalized ambient monitoring that uses mobile technologies to analyze the daily routine and lifestyle of bipolar patients. These studies show that such systems may provide a way of monitoring social activity, relatively unobtrusively, and of following mood variations.

Above and beyond the collection of physiological data (included in ESM), wearable health systems may contribute towards the collection of clinical information and establish a continuous interaction with physicians, for example, via a mood diary posted on a website, software on a computer, or a smartphone application. Indeed, physiological monitoring is now possible using micro-noninvasive biosensors embedded in objects such as bracelets or clothing items and even smart t-shirts [23, 99]. Activation of the monitoring itself can also be done via computer software or a smartphone application [15–20, 100].

#### 3.2.3. European Projects for Personal Health Systems in the Field of Mental Health

The European Commission (EC) supports a number of mental health projects involving personal health systems under the 7th Framework Programme (FP7) [21, 101]. The projects target depression (Help4Mood, ICT4Depression, Optimi), psychosocial stress (Interstress), and bipolar disorder (Monarca, Psyche) and make use of mobile technologies (smartphone or computer), virtual reality (avatar), and biosensors (monitoring) [21, 101]. Help4Mood, Interstress, and Optimi provide a new outlook on the use of new technologies for psychotherapy generally and cognitive behavioral therapy in particular [102–106]. Other projects rely on wearable biosensor devices for monitoring physiological parameters and on electronic self-assessment of mood so that the early warning signs of relapse into depression (ICT4Depression: [107]) or bipolar disorder (Monarca: [45] and Psyche: [22, 108]) can be better recognized.

### 3.3. The Challenges of Multiparametric Evaluation

In recent years the transition from ordinary mobile phones to smartphones has transformed a simple telephone communication tool into a multifunctional object incorporating the possibility of more sophisticated means of communication such as Internet and Bluetooth connections. Furthermore, physiological monitoring has been simplified with features such as smart clothes which integrate very small biosensors and can be used to both multiply the number of parameters that can be measured and introduce physiological and psychological measurements into ambulatory practice. Thus, the progress made in EMA technology and physiological multiparameter monitoring over the past decade now makes it possible to combine these two strategies in a new generation of studies. This new area of medicine, especially in the field of mental health, needs to integrate and combine wireless psychological and physiological monitoring and rethink the roles and contributions expected of patients and clinicians.

## 4. The PSYCHE Project 

The European PSYCHE project associates 10 scientific partners from private industry, fundamental research, and academia (http://www.psyche-project.org/). Its goal is the development of a personal, cost-effective, multiparametric monitoring system based on textile platforms and portable sensing devices for the long-term and short-term acquisition of data from patients affected by mood disorders. The project has developed innovative portable devices for monitoring physiological markers, including voice analysis, and a behavioral index correlated to the patient's clinical state. The data acquired will be processed and analyzed on the dedicated platform so as to check the disease diagnosis and help with the prognosis. Finally, communication and feedback to the patient and physician will take place via a closed-loop approach that will facilitate disease management by fostering new collaboration, affording the patient more independence and empowerment. The physiological data collected with these noninvasive devices will include HRV, respiratory rate, activity, and movement, as well as voice. The devices are already available and have been successfully tested for managing diseases such as heart disease, chronic obstructive pulmonary disease, metabolic disorders, and diabetes.

### 4.1. Selection of Follow-Up Parameters

During the development phase, four criteria (clinical, technical, ethical, and pragmatic) were used to select the parameters. They are based on clinical literature (as reviewed in the first section of this paper) and clinical experiences. The technical criteria are based on feasibility and wearability. For ethical and pragmatic reasons, we were at pains to ensure that the system does not stigmatize patients or constitute an intrusion or constraint with respect to their habits.

### 4.2. General Architecture of the PSYCHE System

**Figure 1:**
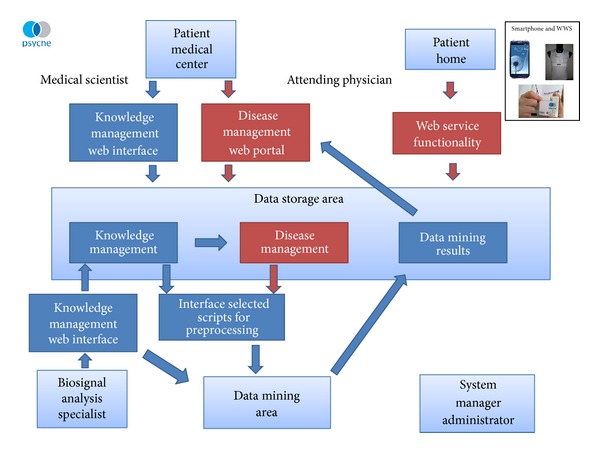
General architecture of data management in the PSYCHE project.

The PSYCHE system consists of two prongs (Figure 1). The first is the knowledge management system. During the preliminary phases of the study, and based on assessments of patients and a controlled use of the system in the research centers, data relating to the clinical characteristics and selected physiological parameters will be collected in a database (see below for the recording system). These data will be used for data mining to try to build algorithms for the ambitious goal of predicting clinical state. The second prong, the disease management system, enables interaction between the physician and the patient. It is reliant on a professional web portal, a platform providing information about the patient's current situation and its evolution, and what the system predicts as to the short-term evolution of the mood status.

The PSYCHE system involves five main contributors: (i) the attending physician, who assesses the patient and together with the patient defines the parameters for the smartphone, (ii) the patient, who is assessed at the medical center and is the user of the system (smartphone, WWS), (iii) the researcher, who downloads measurements and patient data for research purposes, (iv) the biosignal analysis specialist, who is the researcher who designs and analyzes the collected data, and (v) the system manager and administrator who are responsible for the system's functionality and maintenance.

The data recording system is composed of a WWS and a smartphone. The WWS consists of a garment connected to a portable electronic device, the SEW (Side Electronics Wearable), via a standard 4-pin jack connector [109]. The SEW battery has an autonomy of 24 hours, and there is a Bluetooth connection between the SEW and the smartphone. The patient records the data on the smartphone and they are transmitted from the smartphone via the patient's WiFi connection to the PSYCHE database management system.

The PSYCHE garment (a t-shirt for men or women, or a bra for women) is composed of apposite textile interfaces designed to fulfil ergonomic as well as functional criteria to avoid any interference with user activities without invalidating sensor performances. The system design is easily worn as the patients' go about their daily life and during sleep. The aim was to guarantee a high level of comfort so that the patient is motivated to use the system. The sensing platform for use during the acquisition phase has been designed via a cut and sews process. Two fabric electrodes are placed on the patient's thorax, and a multilayer structure is used to increase the pressure and amount of sweat. Finally, the garment with the portable electronic device is able to collect the following parameters: heart rate, breathing rate, breathing amplitude, HRV, posture and/or activity classification (lying, standing, walking, and running), and estimation of energy expenditure [109].

The PSYCHE system can be used to monitor physiological signals during daily activity. In particular during the night, the bra is comfortable, and the electronic portable device is small enough to be worn with the t-shirt in a small pocket on the sternum (the dimensions of the device are 63 mm × 65 mm × 15 mm). The correlation of the different signals provides important information, related to spikes registered by the 3 axis accelerometers, the ECG signal variation, and heart rate actigraphy [22, 23, 109].

The portable electronic device will record the physiological signals on a local memory, which will be downloaded for offline processing.

The smartphone used for the study will be a special one, part of the technological developments of the PSYCHE project. It will include applications responsible for recording a sleep agenda, a mood agenda, and a more detailed clinical assessment of mood state in the form of visual analogue scales from the Bauer Internal State Scale [110]. The smartphone also collects voice samples, elicited through a brief task of comments about pictures presented through the smartphone.

The smartphone will also be used to transmit the data collected from the SEW for sending to the central webserver of the knowledge management system. To reduce the size of the data transmission the data are already stored, calculated, and compressed at harvest before being sent (both by the electronic portable device for the physiological data and by the smartphone for the voice recording).

The smartphone will use a 3G or WiFi communication to send data to the knowledge management system, and then information will be sent to the disease management system. The data are encrypted using secure connections (HTTPS with trusted certificate), and all the information circulated is anonymous. The information on the professional web portal of the disease management system will only be accessible to the physicians and researchers involved in the clinical studies in the context of the PSYCHE project.

**Figure 2:**
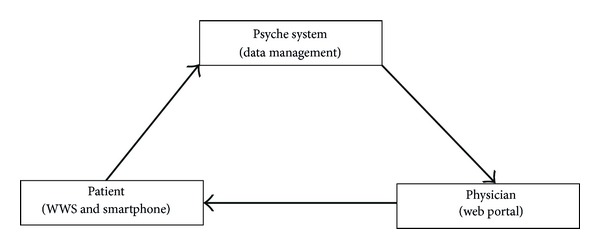
Psyche network.

Figure 1 provides a schematic vision of the general data management architecture of the PSYCHE monitoring system.  Figure 2 summarizes the specific network between the PSYCHE system, physician, and patient.

### 4.3. The PSYCHE Concept

Below is a brief overview of the preliminary studies developed during the first stages of the study.

#### 4.3.1. Preliminary Results about Physiological Data and Sleep Assessment

In a first study [111] the authors analyzed the HRV signal during sleep in one 37-year-old depressed bipolar female patient in different phases of her bipolar disorder and compared the results with those obtained for 8 healthy control subjects. An analysis of HRV signals and movement was used to classify the time spent asleep in 3 phases (wakefulness, rapid eye movements (REM), and non-REM) and to estimate the REM percentage of total sleep time. Recordings of the patient were made during four different nights, at least 1 week apart. The main results showed that some of the parameters examined confirmed reduced HRV in depression and bipolar disorder. The REM percentage was found to be increased. The extraction of reliable features and parameters from the data acquired remotely through wearable platforms was shown to be feasible.

A later analysis of a larger sample [112] confirmed this feasibility. In this second study data from a total of 25 recording sessions involving 12 bipolar patients in different mood states were compared with data from healthy control subjects (102 recording sessions). Once again some parameters of the HRV differed between patients and controls, with some specific characteristics for hypomanic and mixed states on the one hand and for depressed states on the other hand. Sleep-phase information was obtained and showed an increase in REM sleep as a percentage of total sleep time in all patients and a reduction in sleep efficiency in mixed or hypomanic states and shorter REM latency in patients in all states, including the euthymic state.

HRV analysis was taken further in another study [113] which used the HRV data collected from 8 bipolar patients during 3 to 6 recording sessions per patient in four different mood states (euthymic, hypomanic, depressed, or mixed). Based on more than 400 hours of recordings, the authors built a mood state identification paradigm based on an intrasubject evaluation in which the patient's states were modelled as a Markov chain. With this statistical method, each mood state refers back to the previous one. In doing so, the authors were able to attain mood state recognition with an accuracy of up to 99%.

Another study [114] analysed data from voice recordings obtained with 6 bipolar patients during two different mood states and during different tasks (with or without emotional valence) (3 hypomanic and 3 depressed for the first session, all euthymic for the second session). Here, the authors proposed an algorithm based on an automatic voice segmentation of speech signals to detect voiced segments and a spectral matching approach to estimate pitch and pitch changes. Given the limited number of subjects it was not possible to perform a group level analysis but at single-subject levels statistically significant differences were obtained for parameters such as pitch and jitter.

#### 4.3.2. Acceptability and Use of the Wearable Wellness System

During two initial pilot studies, healthy subjects or patients were asked to fill in a questionnaire devised to ascertain the acceptability and usability of the PSYCHE system, since the long-term aim of developing a WWS is to create a new clinical tool that can be used at home.

In the first study, 11 female subjects were included in a clinical study of healthy volunteers with a view to validating the technical device (FORENAP R&D Center, Rouffach, France). They filled in questionnaires on day 1, straight after wearing the WWS bra, and on day 3, just before leaving the study center. Personal use of the system was not tested in this study, since the volunteers did not use it by themselves. The analysis shows that the bra was perfectly tolerated. No difficulties were reported with setting up the system, except for one participant who had difficulty plugging the cable in the SEW. No participants indicated having had problems removing the bra or feeling constricted in their movements during the study. All of the subjects indicated that, based on medical advice, they would agree to wear the bra.

In the other pilot study 5 bipolar patients (4 male and 1 female) were asked to fill in a two-part questionnaire. The first part was filled in when the patient arrived at the study center for a day-time recording period (day 1), and the second part the following morning, after the night sleep spent at home wearing the system (day 2). All five patients indicated that they would agree to wear the WWS following medical advice.

Qualitative feedback (i.e., verbal interactions during various assessments) suggests, however, that patients would not wear the WWS for long periods of time. Their major complaints arguing against long-term use were that the garment was hot to wear and felt “tight.”

It is not surprising that bipolar patients might be slightly more apprehensive than healthy volunteers and might have more difficulty using the WWS. The prospect of wearing a WWS 24 hours a day and 7 days a week is clearly difficult for both volunteers and patients. Its acceptability seems to be related to very practical reasons, namely, the idea of wearing a t-shirt at all times and in all seasons and of always having a clean wearable system to hand.

Following these early results, technological system improvements and adaptations were made, for example, regarding the size and flexibility of the garment. The study consortium was also prompted to abandon the prospect of wearing the WWS most of the time in the next generation of validation studies and to consider more limited use of the system (see below).

#### 4.3.3. Upcoming Clinical Validation Studies

The preliminary studies conducted during the first phase of the PSYCHE project, as described above, were limited to assessing the selected parameters to identify the subject's current mood state. In the next phase of the project, the aim will be to assess the feasibility of using the PSYCHE system to predict significant clinical changes for bipolar patients. These will be exploratory studies, and the number of patients will be limited by the availability of prototypes (the limiting factor being the electronic portable devices rather than the garments or smartphones). In the two studies that are planned each PSYCHE recording with the WWS will be accompanied in parallel by a clinician-based assessment of the patients' depressive and manic symptoms with the QIDS-C16 (16-item Quick Inventory of Depressive Symptomatology—clinician rating) [115] and YMRS (Young Mania Rating Scale) [116]. Moreover, using their PSYCHE smartphone, subjects will send in daily reports of their mood and sleep quality throughout their participation in the study.

The first study will assess whether the PSYCHE system is able to predict how mood states evolve, and whether it can contribute in any way to clinical follow-up within the context of treatment of an acute bipolar episode. Ten in-patients will be recruited in Pisa (Italy), and they will be recorded during a 19-hour session (including during the night) once a week. The maximum duration of the session is determined by the battery life of the electronic portable device. Patients will be studied during clinical remission from the time they are admitted to hospital to the time when they leave.

The second study will address the question of whether the PSYCHE system can detect and predict subjects' mood swings within the bipolar spectrum. To that end, 16 subjects with either a rapid-cycling bipolar disorder or cyclothymic disorder or temperament will be recruited in Geneva (Switzerland) and Strasburg (France). During the 14-week study patients will perform a twice-weekly night recording with the PSYCHE system (including a limited period of time in the evening before going to bed and in the morning after getting up). The clinical profile of subjects was chosen to maximize the number of fresh mood phases during the study. Subjects with cyclothymic disorder or temperament could be particularly interesting in this respect. The subjects will be recruited among both the clinical and the general population.

## 5. Discussion

This paper reviews what we know about objective physical correlates in the field of mood disorders and addresses this question from the perspective of clinical, biological, and physiological markers. Despite the dramatic increase in our knowledge in this field, it has to be said that there is still a lot that we do not know and, more importantly, no biological or physiological marker has yet emerged as a relevant contribution for helping clinicians. There may be several reasons for this. Some may be due to limitations that have to do with the cost or ease of data collection. Others are due to the lack of predictive power of a variable when considered alone. The development of ICTs in the field of health and in particular mood disorders opens up the prospect of integrative approaches whereby multiple variables can be assessed using the highly sophisticated solutions ICTs have to offer. The PSYCHE project as presented in the last section of this paper comes within the context of the ambitious prospect of overcoming the aforementioned limitations. The PSYCHE system offers an easy, comfortable, and user-friendly way of collecting multivariable multiparametric data. Preliminary studies conducted in the framework of the PSYCHE project show the potential of single parameters (such as HRV or voice characteristics) to identify current mood states and the usability and acceptability of the system for patients (even if, following patient feedback, we decided to reduce recording time frequency and duration). The challenge facing the project, however, will be that of going beyond current mood state identification to try to predict short-term mood changes with a view to predicting how patients will respond to either treatment during an acute episode or the recurrence of new episodes.

Such health systems derived from ICTs have the potential to promote deep changes in the way care is organized. Some authors have highlighted the partnership position of patient issues [80] and the relevance of repeated assessments to detect subtle changes [117]. Other authors have stressed that these new developments in the ICT field must take account of the stances and expectations of both patients and clinicians [118, 119] and that the question of potential damage to the therapeutic relationship is one that needs to be addressed [120, 121].

## 6. Conclusion

Thanks to progress these past few decades in the field of communications and the use of biosensors for data capture purposes, it is now possible to consider actively promoting wearable health systems in many different branches of medicine and in particular in the mental health field. For a long time, studies of bipolar disorder have addressed the question of the benefits to be gained from monitoring physiological parameters and mood changes as potential predictors of relapses. There are now many great protocols in place for assessing the benefits of multiparameter monitoring via wearable health systems with respect to the different pathologies encountered along the bipolar disorder spectrum. The way the healthcare system is organised could look very different in the future if a role is assigned to this dynamic form of care that can contribute towards reshaping the relative positions of patients and clinicians and involves giving worthwhile thought to the questions of data security and the ethical dimension to healthcare.

The PSYCHE project is part of the recent development of ICTs in the field of mood disorders. The exact future of this field remains to be seen. It is a way paved with many questions, many risks, many challenges, but also many opportunities. This paper has tried to give the reader an overall view of the current situation and to illustrate potential with a specific project by way of example.
